# Clinical and public health measures to cope with respiratory syncytial virus infection

**DOI:** 10.2478/abm-2025-0014

**Published:** 2025-06-30

**Authors:** Editorial Office of Asian Biomedicine

**Affiliations:** Faculty of Medicine, Chulalongkorn University, Bangkok 10330, Thailand

Respiratory syncytial virus (RSV) is a common respiratory virus that causes seasonal outbreaks of respiratory tract illness worldwide, usually during the winter season. RSV typically produces mild, cold-like symptoms but can lead to severe illness in infants, older adults, and immunocompromised individuals [1]. RSV is the most common etiology of bronchiolitis in children <2 years of age [2]. In immunocompromised patients (adults and children), RSV is also an important and often unrecognized cause of lower respiratory tract infection [3]. RSV reinfection is common throughout life [4]. Effective clinical and public health measures are essential to manage RSV infection, reduce transmission, and prevent complications.

The clinical manifestations of RSV infection vary with age, health status, and whether the infection is primary or secondary. Infants and young children with bronchiolitis typically present with initial upper respiratory symptoms, such as rhinorrhea, followed by lower respiratory tract signs, including wheezing and/or crackles. The clinical spectrum can range from mild symptoms to acute respiratory failure. RSV infection usually is self-limited but has been associated with recurrent wheezing in some patients. Among infants and young children, RSV bronchiolitis should be suspected in infants with compatible clinical and epidemiological features (e.g., age <24 months, respiratory distress with wheezing, winter season, and known circulation of RSV) [5].

Hydration and oxygen therapy are the desirable supportive care for hypoxemia, nasal suctioning for infants with congestion. The efficacy of bronchodilators for bronchiolitis is debatable. Evidence-based recommendations regarding diagnosis, treatment, and prevention were proposed with a high degree of consensus. Although supportive care remains the cornerstone for the management of RSV infections, new monoclonal antibodies, vaccines, drug therapies, and viral surveillance techniques are being rolled out [6]. Preventive measures are important, particularly when RSV infections may be a significant promise, and we are on the cusp of a new era in RSV prevention, which may impact the subsequent development of long-term wheezing and asthma [7].

In this issue, Chantasrisawad N, Boonjindasup W, Polite FG, Puthanakit T, and Chaithongwongwatthana S have carried out a comprehensive review of RSV infection in infancy [8]. Since RSV may have long-term consequences, it is essential to focus on the prevention of unwanted subsequent illnesses. According to the review, recent efforts and advancements have concentrated on preventing severe disease through passive immunization, such as bivalent RSVpreF administered during pregnancy. Immunization can transfer protective antibodies to the infant. The use of long-acting monoclonal antibodies, such as nirsevimab, providing season-long protection with a single dose has also been approved by the Food and Drug Administration [9].

Community-based strategies are recommended to create public awareness (educating caregivers on RSV symptoms and when to seek medical help). The implementation of daycare and school policies aimed at encouraging sick children to remain at home. People are encouraged to practice respiratory etiquette, such as covering coughs/sneezes and proper tissue disposal. During RSV seasons, it is important to institute surveillance and outbreak response measures for RSV. Viral coinfection tracking is recommended to detect the circulation of RSV commonly occurs with influenza and other viruses such as COVID-19 [10]. Broader RSV vaccines for older adults, improved antiviral therapies, and established Global RSV surveillance networks are also needed for early outbreak detection and effective prevention and treatment.
